# Comparison of light transmittance and color changes between polyurethane and copolyester retainer materials after staining and destaining

**DOI:** 10.1186/s12903-024-03887-6

**Published:** 2024-01-31

**Authors:** Grace Viana, Insia Virji, Laurie Susarchick, Veerasathpurush Allareddy, Sullivan Lown, Max Gruber, Henry Lukic, Spiro Megremis, Phimon Atsawasuwan

**Affiliations:** 1 Department of Orthodontics, University of Illinois Chicago, Chicago, IL 60612 USA; 2 Dental Materials & Devices Research - American Dental Association, Chicago, IL 60610 USA

**Keywords:** Orthodontic, Clear retainers, Surface roughness, Copolymer, Transparency, Color stability, Dentistry, Retention

## Abstract

**Background:**

Retainers are the only effective approach to prevent orthodontic relapse. The aim of this study was to compare the changes in color and light-transmittance of rough and smooth thermoformed polyurethane and copolymer retainer samples after staining in different solutions and destaining with different approaches.

**Methods:**

Four hundred copolyester (Essix® ACE) and 400 polyurethane (Zendura®) samples with different surface textures, smooth and rough, were stained in 4 different solutions (*n* = 100 per solution) over 28 days. Each of the four groups of 100 stained samples of each material was subdivided into 5 groups of 20 samples and subjected to different destaining solutions. Light transmittance and color changes were evaluated using a spectrometer and a spectrophotometer. Mean differences were compared using a two-way analysis of variance (ANOVA) and posthoc multiple comparison tests at *P* = 0.05.

**Results:**

No significant differences in light transmittance were found between both untreated materials. Both materials were stained in a similar fashion and showed no significant differences between two materials after staining. Coffee and tea stained both materials more significantly than wine, but there was a significant difference of changes of color and light transmittance between rough and smooth surfaces during the destaining in coffee- and tea-stained samples of copolyester material. All destaining solutions were effective at removing all stains on the samples. The surface roughness of the material plays a significant role in the ability of the materials to be destained, demonstrating a more significant greater effect on cleaning rough samples for improvements in light-transmittance and greater changes in color.

**Conclusions:**

This study concluded that the surface of materials plays a significant role in the material destaining and staining. In addition, the different polymers used for retainer fabrication exhibited different responses during the destaining process depending on types of stains.

**Supplementary Information:**

The online version contains supplementary material available at 10.1186/s12903-024-03887-6.

## Background

Post-treatment relapse is one of the orthodontic complications, which can lead to both time and financial burdens [1]. Orthodontic relapse is defined as teeth returning to their original position after orthodontic treatment [2]. Because orthodontics cannot predict which patients are at risk of relapse, retainers are the only effective approach to prevent orthodontic relapse [2]. There are several types of orthodontic retainers [1]; however, clear thermoformed retainers gain more popularity due to their esthetic appearance and easiness of maintaining periodontal health [3, 4]. To maintain good compliance with clear retainer wear, transparency and color stability of clear retainers need to be maintained, and they are critical considerations for patients and clinicians [5]. Few studies investigated the long-term effectiveness of cleaning solutions for maintaining color stability and light transmittance of clear thermoplastic retainers [6–9]. However, characterizing the ability to remove a “stain” once it has occurred requires further investigation [10].

Several polymers generally used for fabricating clear thermoplastic retainers are polyurethane, polypropylene/polyethylene copolymer, polyethylene terephthalate glycol (PETG) copolyester, polycarbonate and ethylene vinyl acetate [11]. Among these materials, PETG and polyurethane are commonly used for thermoplastic orthodontic retainers [11]. Each polymer possesses biocompatible, chemical, and physical properties suitable for clear thermoplastic retainers [11]. Thermoplastic clear retainers are normally fabricated by thermoforming polymer sheets over either a plaster dental model or a 3D-printed dental model [12, 13]. These dental models possess different own surface textures, which are then transferred to the internal surfaces of the clear thermoplastic retainers during the fabrication [14, 15]. Studies showed that different cleaning solutions affected the mechanical and physical properties of different thermoplastic retainer materials, including light transmittance, surface roughness, and flexural modulus [6–8]. A report showed that the surface roughness of copolyester material plays an important role in its ability to be stained or destained [9]; however, the comparison of stained-retainer thermoplastic materials with different surface roughness has never been studied in the aspect of staining and destaining. Therefore, this study aimed to compare the changes in light transmittance and color between two retainer materials, polyurethane and copolyester polymers, with different surface textures, rough and smooth surfaces after they have been stained and destained in various solutions.

## Methods

**Fig. 1:**
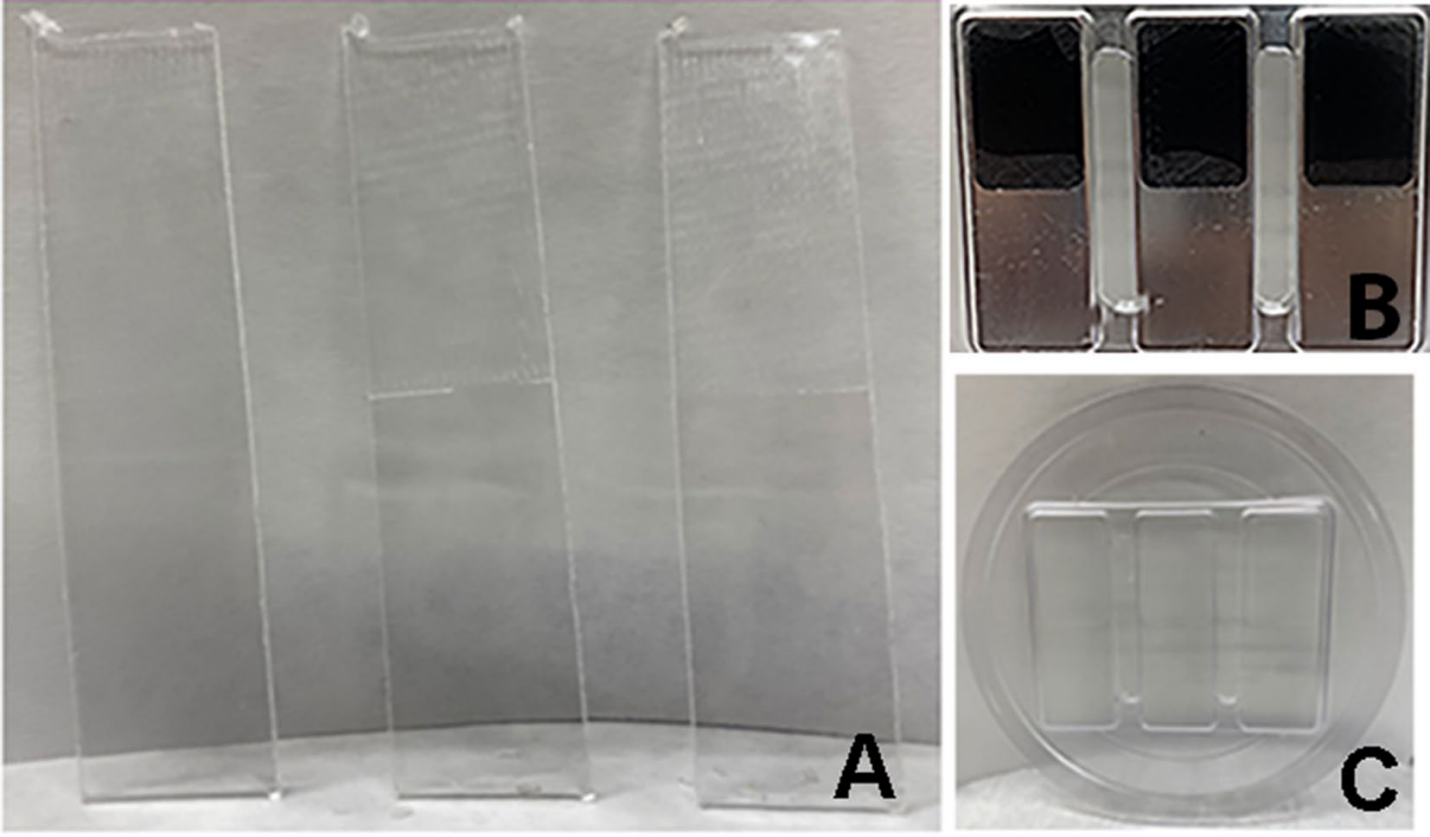
The appearances of samples and their textures and the molds. (**A**) The samples with rough and smooth surfaces. (**B**) The metal molds with textured inserts. (**C**) The thermoformed sheet material with different surface textures after thermoforming

Four-hundred samples of polyurethane [16] (Zendura®, Bay Materials LLC, Fremont, CA) and four-hundred samples of copolyester materials [17] (Essix®ACE, Dentsply Sirona, Inc., Charlotte, NC) were fabricated by thermoforming over aluminum molds (Fig. 1A and B) using the Biostar® machine (Scheu-Dental, Iserlohn, Germany), according to the manufacturer’s instructions. Three samples were obtained and cut from the thermoformed sheets with dimensions of approximately 50.8 mmx12.7 mmx 1.0 mm. This dimension is recommended by (ASTM D 790) “Standard Test Methods for Flexural Properties of Unreinforced and Reinforced Plastics and Electrical Insulating Materials”, which provides for alternative test specimen sizes for materials that are less than 1.6 mm (1/16 in) thick. The rectangular aluminum molds had pockets for textured acrylonitrile butadiene styrene (ABS) printed inserts, representing the surface roughness of the internal surface of Vivera® retainers (Align Technology, San Jose, CA) as approximately half of the length of the thermoformed samples, as shown in Fig. 1A. The average area roughness value (Sa) of the ABS inserts was ~ 10.5 μm with a coefficient of variation (c.o.v.) of 3%, measured by a Zygo New View 8300 optical interferometer (Zygo Corp, Middlefield, CT). The selected Sa value for the inserts was in the range of Sa values of internal surfaces of Vivera® retainers (10.6 ± 9.1 μm with c.o.v. of 86%).

The four hundred samples of each material were divided into four groups of 100 subgroup samples, with each group exposed to a different staining solution. The staining solutions were coffee, black tea, and red wine based on their capability to stain clear retainers and distilled water (control) [9]. Based on previous publication references [6–8], a representative sample with at least 20 units of analysis would yield approximately 80% of statistical power to detect the mean effects of interest at 5% type I error level.

The coffee solution was prepared by mixing 688 g of instant coffee powder (Nescafe® Original, Nestle Ltd. Vevey, Switzerland) into 8 L of distilled water (ISO grade 3), according to the manufacturer’s instructions. The black tea solution was prepared by mixing 150 g of instant tea powder (Lipton® Unsweetened Black Tea Mix, Nestle Ltd., Vevey Switzerland) into 8 L of distilled water (ISO grade 3), according to the manufacturer’s instructions [9, 10]. The red wine was a Cabernet Sauvignon (Paint Box® Cabernet, Columbia Valley, CA). Distilled water (ISO grade 3) was used as a control solution.

The samples were submerged in the freshly made staining solutions at 37 °C and replaced daily for 28 days. Color and percent light transmittance measurements were performed at day 0 (baseline), 14, and 28. After staining for 28 days, each group of 100 stained samples was divided into five small subgroups of 20 samples each for the destaining experiments. The samples were then subjected to five cleaning solutions: Invisalign® Cleaning Crystals (Align Technology, San Jose, CA), Retainer Brite® (Dentsply, Sarasota, FL), Polident® (GlaxoSmithKline, Research Triangle Park NC), Listerine® mouthwash (Johnson & Johnson, Skillman, NJ), and 3% hydrogen peroxide (H_2_O_2_) stirring with a magnetic stirrer [6–8]. The composition of each cleaning solution was described in Table 1S. The stained samples were destained in the cleaning solutions in groups of 20 for 15 min each, except for the stained samples in the Polident® solution, which were soaked for 3 min, as specified in the manufacturers’ instructions. The samples were kept in artificial saliva [18] at 37 °C between measurements.

**Fig. 2:**
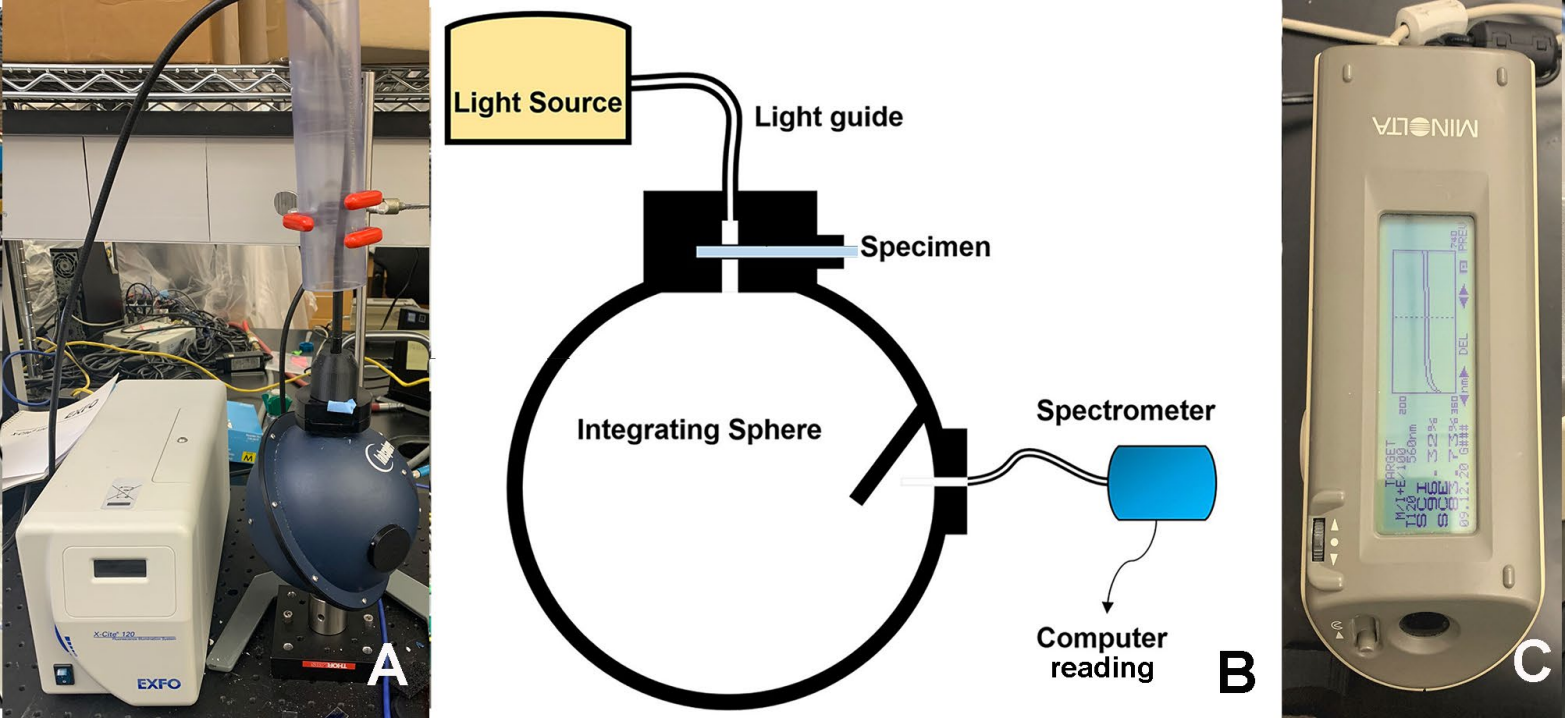
Instruments used for the measurement of studied parameters. (**A**) Spectrometer/Integrating sphere system for evaluation of percent light transmittance, (**B**) A diagram of light transmittance measurement system. (**C**) The Spectrophotometer (CM-2600d Spectrophotometer, Konica Minolta, Tokyo, Japan) for color parameter change

Absolute percent light transmittance was measured as previously published methods for measuring the translucency of retainer materials [6–8]. Briefly, the percent of light transmittance through the retainer material was measured with a spectrometer-integrating sphere system. The percentage of light transmittance through the sample is calculated for wavelengths between 380 and 780 nm (Oceanview software, version 1.5, Ocean Optics, Dunedin, FL, USA) (Fig. 2A and B). Changes in percent light transmittance, ∆T, were calculated for days 14 and 28 with respect to baseline. Changes in percent light transmittance, ∆T, were also calculated following the destaining experiments, comparing light transmittance values at day 28 of staining experiments with the values measured after the respective cleaning methods.

Color changes of the samples were evaluated according to the International Commission on Illumination L*a*b* (CIELAB) color space [19] using a Konica Minolta CM-2600d Spectrophotometer (Konica Minolta Sensing Americas, Inc, Ramsey, NJ) (Fig. 2c). In this color space, L* is a measure of lightness from 0 (black) to 100 (white), and a* and b* are chromaticity coordinates: +a* is red direction; -a* is green direction; +b* is yellow direction; and -b* is blue direction. At each study time point, the sample was measured in triplicate at the same position, and average values were calculated and recorded. Using the CIELAB color space, the color difference (∆E*) was calculated using the following equation [20]:

∆E* = [(∆L*)2 + (∆a*)2 + (∆b*)2]½.

The National Bureau of Standards (NBS) system was used to describe color change as follows [21]:

NBS = [(∆E* at specific day of measurement - ∆E* at measurement baseline) × 0.92]

NBS values above 3.0 were considered marked changes in color [10], which for this study was considered clinically unacceptable (Table 1) [22].

### Statistical analysis

Based on the Shapiro-Wilk Normality test, appropriate statistical analyses were appropriately used for testing group differences. A two-way analysis of variance (ANOVA) was completed to identify inter-material statistical differences (*P* < 0.05) in staining and destaining methods. Estimated marginal means testing was completed to identify statistical differences between groups. Paired t-tests were used to determine intra-material statistical differences (*P* < 0.05) of surfaces and time, and ANOVA with post-hoc Bonferroni tests were used to determine intra-material statistical differences (*P* < 0.05) of stainings and destaining methods. The statistical analysis was performed with IBM SPSS Statistics (version 28; IBM, Armonk, NY).

## Results

No differences of the percent of light transmittance were found between polyurethane and copolyester materials, with rough or smooth surfaces, at naïve stages (day 0) (Table 2S) with p-values > 0.05. After staining, there were no statistically significant mean differences between polyurethane and copolyester materials in the presence of the surface, time, and stains on the changes in percent light transmittance (∆T); F(1,159) = 0.001, p-value = 0.0925 and color (NBS values); F(1,159) = 0.001, p-value = 0.941. Table 2 shows the descriptive and statistical analyses of ∆T for both polyurethane and copolyester materials with different surface textures of three staining solutions and distilled water as the control at days 14 and 28. Table 2 shows that the rough and smooth samples of both studied materials were significantly stained by the coffee and tea compared to wine and water, with respect to ∆T values at day 28. The difference in surface texture exhibited no effect on ∆T of both material samples. However, the significant differences of ∆T between days 14 and 28 were observed only in the coffee-stained and tea-stained in both material samples.

**Fig. 3:**
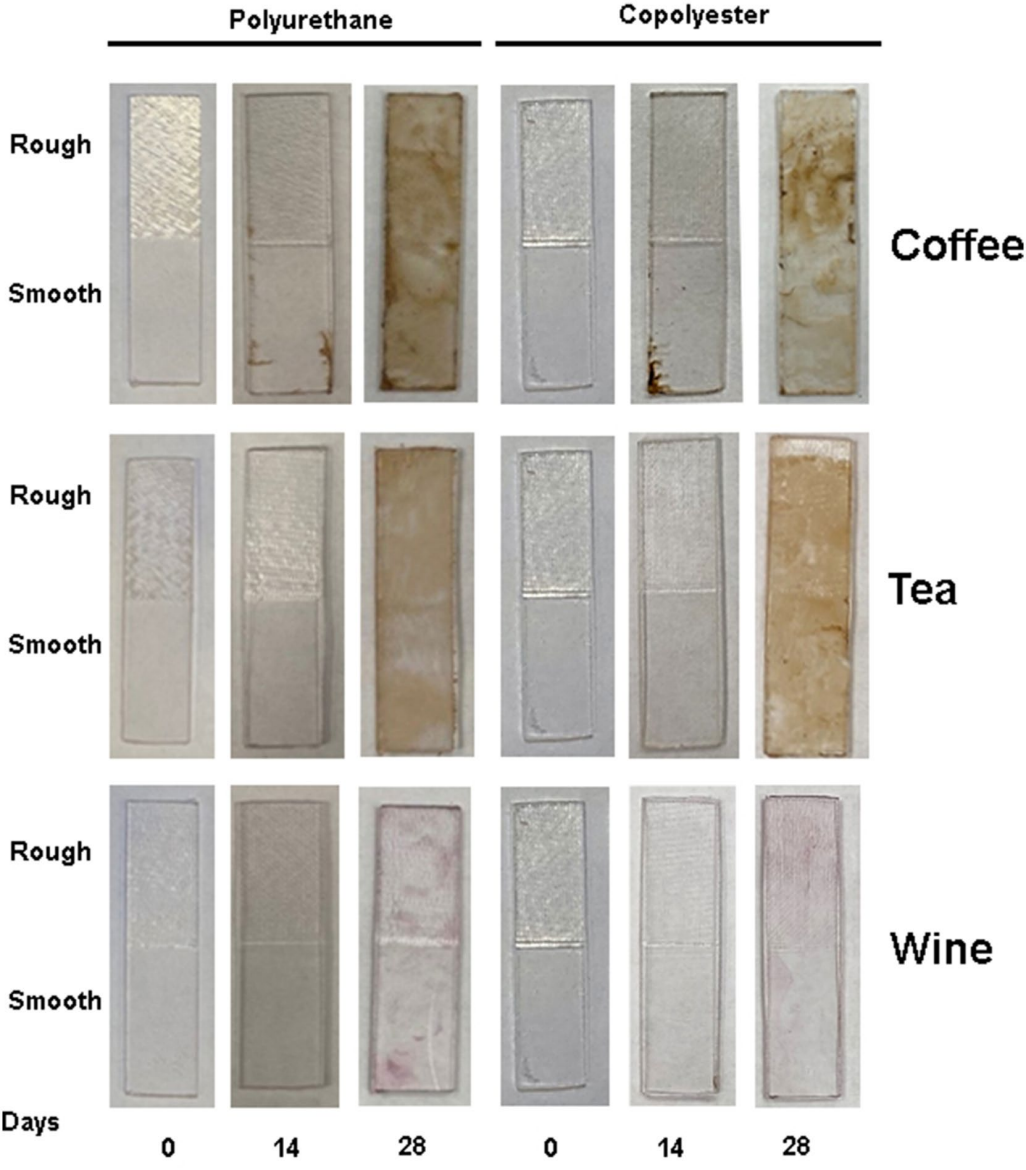
Representative examples of the polyurethane and copolymer retainer materials after staining by the different staining solutions at different time points

Table 3 shows the descriptive and statistical analyses of NBS for both polyurethane and copolyester materials with different surface textures in three staining solutions and distilled water as the control at days 14 and 28. Table 3 shows that the rough and smooth samples of both studied materials were significantly stained by the coffee and tea compared to wine and water, with respect to NBS at day 28. Note that the effects of surface texture were observed only in the copolyester group on day 14. In addition, the significant differences of ∆T between days 14 and 28 were observed only in the coffee-stained and tea-stained in both material samples and the wine-stained polyurethane group. The result is also shown qualitatively in Fig. 3.

After 28 days of staining, each group of stained samples was subjected to different destaining processes. However, after 28 days, since only the samples stained in the coffee, tea, and wine solutions showed significant staining with respect to changes in both ∆T and NBS values compared to the control samples in water (Fig. 3), only the results and analyses for the cleaning methods on the coffee, tea, and wine-stained samples are presented.

There were no statically significant mean differences between polyurethane and copolyester materials on ∆T; F(1,599) = 2.146, p-value = 0.143; however, a statistically significant NBS value was observed between the two studied materials; F(1.5990 = 17.961, p-value < 0.001 during the destaining period. After detaining, polyurethane samples exhibited better improvement of NBS values than those of polyester samples. The surface roughness significantly affects the destaining of both materials in all stained groups with respect to the NBS values. Still, only the coffee-stained group in respect to the ∆T The details of this effect are shown in Tables 4 and 5. For all 5 destaining solutions; these tables show that the rough samples were cleaned to a significantly greater extent than the smooth samples with respect to greater changes in both ∆T and NBS values.

To emphasize the details of the capability to be destained between two studied materials, for instance, for the rough samples stained with coffee, all 5 destaining solutions resulted in changes in percent light transmittance values of about 22–23% in polyurethane and copolyester groups, and for the smooth samples stained in coffee, all 5 destaining solutions resulted in ∆T values of about 15–16% in polyurethane and copolyester groups, (Table 4). Additionally, for the samples stained with tea, though there were no statistical differences, all 5 destaining solutions resulted in percent light transmittance with the ∆T values for rough samples improving by about 19% in polyurethane and 24% in copolyester groups and for the smooth samples by 19% in polyurethane and about 21% in copolyester groups (Table 4). For the samples stained with wine, all 5 destaining solutions resulted in similar changes in percent light transmittance, with the ∆T values for rough samples improving by about 0.5% in polyurethane and 1.5% in copolyester groups and for the smooth samples by about 1% in polyurethane and copolyester groups. No difference in destaining effectiveness among solutions was detected.

For the results of the color change of the samples after application of the destaining solutions, for the rough samples stained with coffee, all 5 destaining solutions demonstrated NBS values of about 19 in polyurethane and 15 in copolyester groups with less effectiveness of H_2_O_2_ (Table 5). Likewise, for the smooth samples stained with coffee, all 5 destaining solutions resulted in NBS values of about 13, with less effectiveness in the samples destained in H_2_O_2_ in polyurethane and about 11 in the copolyester. For the rough samples stained with tea, all 5 destaining solutions demonstrated NBS values of about 16 in polyurethane and 15 in copolyester groups, with less effective in the samples destained in H_2_O_2_; while, for the smooth samples stained with tea, all 5 destaining samples demonstrated NBS values of about 18 in polyurethane and 13 in copolyester groups with less effective in the samples destained in H_2_O_2_ (Table 5). Furthermore, for the rough samples stained with wine, all 5 destaining solutions demonstrated NBS values of about 2.5 in rough and smooth polyurethane and rough copolyester samples and about 1.5 in rough copolyester samples (Table 5). No difference was found among the solutions.

## Discussion

It has been well accepted that long-term retainer wear is the only effective approach to prevent post-treatment orthodontic relapse [1, 2, 23]. Since plaque and calculus buildup and retention can occur on clear retainer material in the oral environment, the clear retainers must be cleaned to delay the accumulation of deposits, which decrease their light transmittance and maintain color stability and material integrity [24, 25]. In addition, several factors, i.e., ultraviolet radiation, food colors, and various beverages, can affect the color stability and transparency of the clear retainers [10, 26]. Different cleaning methods also affected the mechanical and physical properties of the retainer materials, especially the light transmittance over time [6–8].

The polymers we compared in this study were polyurethane and copolymer materials commonly used for the fabrication of thermoformed orthodontic retainers. We showed no differences in the percent of light transmittance between the materials at their naïve stage, so we can further compare their capability of being stained and destained with different solutions. Different thermoplastic materials might react differently when exposed to staining and destaining solutions [6–8, 27]. For example, the acidic nature of wine and coffee can cause surface roughening, leading to easy staining. Tannic acid found in tea and coffee was reported to cause yellow-brown color associated with both absorption and adsorption of the ingredient [28]. A report show that red wine can cause severe staining on certain provisional resin materials [29, 30]. In addition, tea was reported to cause discernible extrinsic stains on the surfaces of aligners, though they were easily removed [10]. In this study, coffee and tea significantly affect the materials more than wine. In the United States, 50% of Americans over 18 years old drink an average of 3 cups of coffee per day in 2023. The top countries in the world with the highest amount of coffee consumption include Finland, Sweden, Switzerland, Germany, France, Italy, Brazil, the United States, Japan, and the United Kingdom [31]. Tea is the most widely consumed beverage globally. In 2020, global tea consumption amounted to about 6.3 billion kilograms. According to the 2016 report on per capita tea consumption, Turkey leads the countries drinking the most tea in the world, followed by Ireland, and the United Kingdom, respectively [32]. Providers should instruct patients to remove retainers before drinking certain staining drinks in addition to eating, as staining could occur from several types of drinks that would allow the stains to accumulate on the clear retainer [10]. This study showed no differences between stained materials with either coffee, tea, or wine, although coffee and tea tended to stain both retainer materials significantly more than wine.

Previous research has shown that different retainer materials exhibited different responses in changes of light transmittance after exposure to different cleaning agents [6–8]. The selected cleaning/destaining solutions in this study were screened according to the previous studies [6–8]. The chosen cleaning solutions showed only minimal effects on the changes in percent light transmittance values compared to baseline values of the retainer samples. Moreover, Invisalign Cleaning Crystal, Retainer Brite, and Polident are commercially available and commonly used for cleaning orthodontic retainers. H_2_O_2_ is also a common ingredient in many commercial aligner cleaning products. Note that all stained samples were exposed to Polident only 3 min per manusfacturer’s instruction while to other solution for 15 min. All destaining solutions in this study were demonstrated to be effective and comparable at removing coffee and tea stains from both materials with respect to increasing percent light transmittance and significantly changing their color. Types of polymers also affected the ability of retainer materials to be destained. Polyurethane was destained easier than copolyester materials, which may be due to the change of endogenous properties of each polymer when exposed to different cleaning solutions [6] or the hydrophilic properties of polyurethane material [33].

An interesting finding of this study is that the surface roughness of the retainer material plays a significant role in the capability of materials to be stained and destained. It was shown that coffee, tea, and wine-stained rough surface samples were more significant than the smooth surface samples affected by more remarkable changes in percent light transmittance and NBS values. It was also shown that all of the destaining solutions had a more significant effect on cleaning the rough samples with respect to improved changes in percent light transmittance and more significant changes in color. A study was reported on the influence of surface roughness on the staining and destaining of copolyester retainer material. The results showed no differences of the staining effect between rough and smooth surfaces. However, there were significant differences among destaining processes between rough and smooth surfaces [9], implicating the crucial role of surface roughness in the destaining of a retainer material. A clinically relevant aspect of surface roughness on different retainer materials should be considered when used clinically regarding the staining and destaining of the materials. Additionally, as previously done for other resin materials, the effect of other factor such as acidic drinks or food and endogenous aging of the materials should be tested on these retainer materials to comprehensively understand the behavior of these materials [34, 35].

## Limitations

This study has some limitations. First, the samples were prepared according to the recommendation of Standards so the samples of different materials can be compared to each other. The samples were not in the arch forms to imitate the clinical situation. The staining procedures were designed to control the length and amount of stains used in the study. The staining was not able to imitate the actual temperature of hot beverages such as hot coffee or tea.

## Conclusions

The surface roughness of retainer material plays a critical role on the ability of the cleaning solutions to remove stains on the materials demonstrating a more significant effect on cleaning the rough samples for improved changes in percent light transmittance and more significant color changes. In addition, the different polymers used for retainer fabrication exhibited different responses during the destaining process depending on the types of stains.

**Table 1:** Criteria of the National Bureu of Standards (NBS) units. Note that a value above 3 is considered of clinical significance in the study

NBS nits	Critical Remarks of Color Differences	
0-0.5	Trace	Extremely slight change
0.5-1.0	Slight	Slight change
1.5-3.0	Noticeable	Perceivable
3.0–6.0	Appreciable	Marked change
6.0–12.0	Much	Extremeley marked change
12.0 or more	Very much	Change to orther color

**Table 2:** Descriptive values of ∆T of the retainer materials with different surface textures at difference times after staining – Mean ($\pm$SD)

Materials	Polyurethane				Copolyester			
Time by Surface and Staining	Day 14		Day 28		Day 14		Day 28	
	Rough	Smooth	Rough	Smooth	Rough	Smooth	Rough	Smooth
Coffee	1.519 (0.690)	1.770 ^b,c^ (0.210)	24.198 ^a,b,d^ (6.224)	16.528 ^a,b,d^ (5.422)	1.220 (0.929)	2.101 ^a,b,c^ (0.258)	23.466 ^a,b,d^ (6.543)	17.429 ^a,b,d^ (7.931)
Tea	0.952 ^a^ (0.407)	0.901 ^a^ (0.291)	20.183 ^a,b,d^ (5.282)	20.368 ^a,b,d^ (5.882)	1.421 (0.656)	1.190 (0.231)	25.190 ^a,b,d^ (2.162)	19.184 ^a,b,d^ (6.160)
Wine	1.055 ^a^ (0.356)	1.130 ^a^ (0.165)	1.176 (1.098)	1.563 (0.711)	1.194 (0.404)	1.051 (0.305)	2.281 ^a,d^ (1.654)	1.926 ^d^ (0.472)
Water	2.285 (0.459)	1.964 (0.258)	2.567 (0.439)	2.276 (0.533)	0.405 (0.590)	0.697 (0.264)	0.154 (0.674)	0.812 (0.281)

∆T = (T at baseline – T at specific day of measurement)

^a^significant difference from water at specific day; ^b^significant difference from wine at specific day; ^c^significant difference from tea at specific day; ^d^significant difference from day 14

**Table 3:** Descriptive values of NBS of the retainer materials with different surface textures at difference times after staining – Mean ($\pm$SD)

Materials	Polyurethane				Copolyester			
Time by Surface and Staining	Day 14		Day 28		Day 14		Day 28	
	Rough	Smooth	Rough	Smooth	Rough	Smooth	Rough	Smooth
**Coffee**	1.191 (0.215)	1.325 (0.332)	19.583 ^a,b,d^ (4.173)	13.711 ^a,b,d^ (4.687)	2.197 ^e^ (0.798)	3.478 ^a,b,c,e^ (0.634)	16.097 ^a,b,d^ (4.283)	12.793 ^a,b,d^ (5.228)
**Tea**	0.870 ^b^ (0.221)	0.962 (0.273)	16.410 ^a,b,d^ (5.092)	18.249 ^a,b,d^ (5.669))	2.957 ^a,e^ (0.447)	2.250 ^a,b,e^ (0.110)	17.157 ^a,b,d^ (3.918)	14.482 ^a,b,d^ (5.024)
**Wine**	1.359 ^a^ (0.254)	1.085 (0.153)	3.138^d^ (1.264)	2.892^d^ (0.547)	2.170 (0.622)	1.438 (0.264)	3.845 ^a^ (1.535)	2.458 ^a,d^ (0.712)
**Water**	0.829 (0.208)	0.888 (0.312)	1.209 (0.221)	1.433 (0.155)	1.268 (0.446)	1.246 (0.233)	0.925 (0.193)	0.837 ^d^ (0.171)

NBS = [(E at specific day of measurement - E at baseline) * 0.92]

^a^significant difference from water at specific day; ^b^significant difference from wine at specific day; ^c^significant difference from tea at specific day; ^d^significant difference from day 14; ^e^significant difference between surfaces at specific day

**Table 4:** Descriptive values of ∆T of the retainer materials with different surface textures, types of stains and cleaning solutions – Mean (SD)

Materials	Stains by Surface and Cleaning Solutions	Coffee		Tea		Wine	
		Rough	Smooth	Rough	Smooth	Rough	Smooth
**Polyurethane**	**Invisalign**® **Crystals**	23.197^a^ (0.608)	15.521 ^a^ (0.327)	19.638 (0.552)	19.224 (0.471)	0.701 (0.755)	1.107 (0.764)
	**Retainer Brite®**	23.523^a^ (0.428)	15.515^a^ (0.537)	19.956^a^ (0.780)	19.238^a^ (0.289)	0.838 (0.860)	0.820 (0.214)
	**Listerine**® **Mouthwash**	23.556^a^ (0.270)	15.538^a^ (0.289)	19.452 (0.887)	19.454 (0.561)	0.453 (0.675)	0.804 (0.274)
	**Polident**®	23.095^a^ (0.720)	15.599^a^ (0.845)	19.557 (1.022)	19.236 (0.402)	0.514 ^a^ (0.655)	1.020 ^a^ (0.349)
	**H**_**2**_ **O**_**2**_	22.769 ^a^ (0.870)	15.079 ^a^ (0.909)	19.788 (1.232)	19.679 (0.816)	0.476 ^a^ (0.482)	0.981 ^a^ (0.564)
**Copolyester**	**Invisalign**® **Crystals**	22.53 ^a^ (1.094)	16.262 ^a^ (0.285))	24.108 (0.824)	21.715 (3.8665	1.270 (0.819)	1.088 (0.584)
	**Retainer Brite®**	22.961^a,d^ (0.418)	16.072 ^a^ (0.544)	24.188 (0.643)	21.452 (3.873)	2.100 (0.931)	1.174 (0.396)
	**Listerine**® **Mouthwash**	21.931 ^a,c^ (0.622)	15.614 ^a,b^ (0.565)	24.031 (1.941)	20.981 (4.380)	1.681 (1.043)	0.758 (0.534)
	**Polident**®	22.084 ^a^ (0.837)	15.734 ^a,b^ (0.317)	23.633 (0.988)	21.902 (4.222)	1.395 (0.587)	1.044 (0.280)
	**H**_**2**_ **O**_**2**_	22.098 ^a^ (1.648)	15.618 ^a,b^ (0.502)	23.446 ^b,c,d^ (4.087)	20.808 (4.251)	1.576 ^a^ (0.667)	0.944 ^a^ (0.277)

∆T = (percent light transmittance after destaining- percent light transmittance before destaining)

^a^significant difference between surfaces: *P* < 0.05; ^b^significant difference between solution and Invisalign crystals: *P* < 0.05; ^c^significant difference between solution and Retainer Brite: *P* < 0.05; ^d^significant difference between solution and Listerine: *P* < 0.05; ^e^significant difference between solution and Polident: *P* < 0.05

**Table 5:** Descriptive values of NBS of the retainer materials with different surface textures, stains and cleaning solutions – Mean (SD)

Materials	Stains by Surface and Cleaning Solutions	Coffee		Tea		Wine	
		Rough	Smooth	Rough	Smooth	Rough	Smooth
**Polyurethane**	**Invisalign**® **Crystals**	19.277^a^ (0.263)	13.443^a^ (0.288)	16.447^a^ (0.248)	18.169^a^ (0.237)	2.537 (0.480)	2.405 (0.368)
	**Retainer Brite®**	19.485^a^ (0.208)	13.381^a^ (0.405)	16.380^a^ (0.230)	18.145^a^ (0.262)	2.489 (0.331)	2.191 (0.312)
	**Listerine**® **Mouthwash**	19.360^a^ (0.283)	13.287^a^ (0.306)	16.415^a^ (0.304)	18.174^a^ (0.171)	2.320 (0.302)	2.415 (0.261)
	**Polident**®	19.311^a^ (0.297)	13.270^a^ (0.313)	16.401^a^ (0.160)	18.144^a^ (0.331)	2.510 ^a^ (0.248)	2.223 ^a^ (0.332)
	**H**_**2**_ **O**_**2**_	18.854^a, b, c, d, e^ (0.477)	12.796^a, b, c, d, e^ (0.339)	16.272^a^ (0.183)	17.969^a^ (0.247)	2.456 (0.246)	2.255 (0.194)
**Copolyester**	**Invisalign**® **Crystals**	15.107 ^a^ (0.372)	11.013 ^a^ (0.182)	15.724 ^a^ (0.292)	13.502 ^a^ (0.161)	2.757 ^a^ (0.331)	1.729 ^a^ (0.145)
	**Retainer Brite®**	15.024 ^a^ (0.255)	10.983 ^a^ (0.285)	15.720 ^a^ (0.289)	13.170 ^a^ (0.403)	2.701 ^a^ (0.297)	1.741 ^a^ (0.144)
	**Listerine**® **Mouthwash**	14.539 ^a^ (0.496)	10.697 ^a^ (0.468)	15.433 ^a^ (0.714)	13.175 ^a,b^ (0.284)	2.684 ^a^ (0.330)	1.782 ^a,e^ (0.174)
	**Polident**®	14.722 ^a^ (0.343)	10.822 ^a^ (0.453)	15.731 ^a^ (0.268)	13.350 ^a^ (0.177)	2.829 ^a^ (0.336)	1.550 ^a,d^ (0.157)
	**H**_**2**_ **O**_**2**_	14.169 ^a,b,c^ (0.670)	10.580 ^a^ (0.251)	14.591 ^a,b,c,e^ (0.913)	12.689 ^a,b,c,e^ (0.754)	2.752 ^a^ (0.269)	1.551^a^ (0.251)

NBS = [(E at specific day of measurement - E at baseline) * 0.92]

^a^significant difference between surfaces: *P* < 0.05; ^b^significant difference between solution and Invisalign crystals: *P* < 0.05; ^c^significant difference between solution and Retainer Brite: *P* < 0.05; ^d^significant difference between solution and Listerine: *P* < 0.05; ^e^significant difference between solution and Polident: *P* < 0.05

### Electronic supplementary material

Below is the link to the electronic supplementary material.

Supplementary Material 1

## Data Availability

The datasets used and/or analyzed during the current study are available from the corresponding author on reasonable request.

## Abbreviations

PETG: Polyethylene Terephthalate Glycol
COV: Coefficient of Variation
ABS: Acrylonitrile Butadiene Styrene
H_2_O_2_: Hydrogen Peroxide
CIELAB: International Commission on Illumination L*a*b*
NBS: National Bureau of Standards
ANOVA: Analysis of variance
