# Supplementary material for: Comparison of light transmittance and color changes between polyurethane and copolyester retainer materials after staining and destaining
Source: BMC Oral Health. 2024 Jan 31;24:144. doi: 10.1186/s12903-024-03887-6 (PMC10829172; doi:10.1186/s12903-024-03887-6)
Supplement: Supplementary file 1 — Supplementary Material 1 [file 12903_2024_3887_MOESM1_ESM.docx]

**Table 1s. Compositions of each cleaning solution in the study.**

| **Name** | **Compositions** |
| --- | --- |
| **Invisalign crystal®** | Sodium Sulfate, Sodium Carbonate, Sodium Tripolyphosphate, Sodium Dichlorosocyanurate, Sodium Lauryl Sulfate |
| **Retainer Brite®** | Potassium Monopersulfate, Sodium Perborate  Monohydrate, Sodium Sulfate, sodium carbonate, Sodium Bicarbonate, Sodium Tripolyphosphate, Sodium Lauryl  Sulfoacetate, Ethylenediaminetetraacetic  acid, tetrasodium |
| **Listerine® mouthwash** | Ethyl alcohol (denatured) |
| **Polident®** | Sodium carbonate, Sodium Perborate Monohydrate, Potassium Peroxymonosulfate, Citric acid, Sodium Benzoate, Sodium Lauryl Sulfoacetate, Subtilisin, Pepermint Oil |
| **3% Hydrogen peroxide** | Hydrogen Peroxide, Water |

**Table 2s. Values of percent light transmittance (Mean+SD) of the retainer materials at naïve stage**

| **Polyurethane** | | **Copolyester** | |
| --- | --- | --- | --- |
| **Day 0 (mean, +/- standard deviation)** | | **Day 0 (mean, +/- standard deviation)** | |
| **Rough** | **Smooth** | **Rough** | **Smooth** |
| 89.467 (0.943) | 89.874 (1.990) | 90.608 (0.847) | - 1. (0.455) |
